# Effects of Leader Narcissism on Career Success of Employees: An Interpersonal Relationship Perspective

**DOI:** 10.3389/fpsyg.2021.679427

**Published:** 2021-12-20

**Authors:** Huaqiang Wang, Dan Li, Lei Wu, Zhihui Ding

**Affiliations:** ^1^School of Economics and Management, Yangtze University, Jingzhou, China; ^2^School of Business Administration, Zhongnan University of Economics and Law, Wuhan, China; ^3^Business College of Shaoxing University, Shaoxing, China; ^4^School of Law and Public Administration, China Three Gorges University, Yichang, China

**Keywords:** leader narcissism, career success, relationship conflict, dominant personality traits, interpersonal complementarity theory

## Abstract

Previous studies have shown that leader narcissism has a significant impact on the effectiveness of a leader and employee behaviors; however, research on career outcomes of employees is still inadequate. This study explores the effects of leader narcissism on the career success of employees from an interpersonal relationship perspective and examines the mediating role of supervisor-subordinate conflict and the moderating role of dominant personality traits of employees. Data from 291 employees in Chinese companies have revealed that leader narcissism, directly and indirectly, affects the career success of employees through supervisor-subordinate relationship conflict. However, dominant personality traits of employees strengthen the impact of leader narcissism on supervisor-subordinate relationship conflict. The theoretical and practical implications of the findings of this study are further discussed.

## Introduction

There is a growing acknowledgment that career success is the main driving source of the behavior of employees at the workplace (Spurk et al., 2019). Career success of individuals not only provides opportunities for promotion and salary increase but also has a close relationship with the realization and maintenance of his/her self-worth (Arthur et al., 2005). Defined as the accumulation of positive psychological or work-related outcomes resulting from the work experiences of individuals, career success includes both subjective (e.g., career satisfaction) and objective (e.g., salary attainment and number of promotions) components (Seibert et al., 2001). However, pursuing career success is not always smooth sailing; various challenges must be overcome (Guan et al., 2019). The accomplishment of an employee represents his/her crucial contributions to achieve the goals of an organization and shows the effectiveness of behaviors of his/her leader (Ng et al., 2005). Thus, career success has become an inevitable issue that needs to be considered in the workplace. If an employee cannot achieve his/her career success regardless of how much effort he/she puts into his/her work, he/she will be more likely to intend to leave his/her position, resulting in potential management costs and risks to the organization (Spurk et al., 2019). Therefore, the antecedents of career success have been extensively studied to reconcile this issue.

As the agent of organizations, leaders are responsible for resource allocation and promotions of employees. Without support from the immediate supervisor, the employees will face considerable obstacles in their career development (Astakhova, 2016). Previous research has shown that a leader plays a vital role in the career success of employees. However, most studies focus on the impact of behaviors of a leader (Wayne et al., 1999; Ng et al., 2005). However, less research is available regarding investigating the impact of personality traits of a leader of which the critical role was highlighted in managing the employees (Salas Vallina et al., 2019). Unlike leader behaviors, his/her traits are relatively stable with longer-lasting effects on employees (Judge et al., 2009). Therefore, researchers have recently started exploring the relationships between personality traits and career success of leaders (Chang et al., 2020; Rigotti et al., 2020). Besides, some scholars have called for a need to explore the outcomes of dark traits (e.g., narcissism) in leadership contexts (Judge et al., 2009; Braun, 2017). Leader narcissism, characterized as a sense of grandiosity and an inflated self-view and tendency to act in self-interest, is a typical leader dark trait (Grijalva et al., 2015). Previous research has linked leader narcissism to several bad work-related outcomes (Braun et al., 2018; Liao et al., 2019). However, few studies have empirically explored the impact of leader narcissism on the career success of employees (Volmer et al., 2016). This is somewhat surprising because prior research believes that leader narcissism could be leading in an autocratic, inconsiderable, exploitative, and self-serving manner (Grijalva et al., 2015), thereby hard for subordinates to acquire resources to facilitate their professional career. Thus, it is of high value to investigate the influence of leader narcissism on the career success of employees.

To better understand the relationship between leader narcissism and career success, this study seeks to explain it by applying the interpersonal relationship perspective, which is widely used to explore the influence of leadership on employee outcomes (Erdogan et al., 2015). Being one of the most crucial factors of interpersonal relationship perspective, supervisor-subordinate relationship conflicts are considered costly, yet inevitable elements of work relationships, and have been related to adverse employee outcomes (Jehn et al., 2010; Tillman et al., 2017). Stemming from an interpersonal relationship perspective, subordinate employees have less confidence in their work and do not obtain resources due to conflict with their supervisor than their counterparts; consequently, it is hard to realize a successful career (Xin and Pelled, 2003). Furthermore, from an interpersonal relationship perspective, different individuals will have different reactions in the face of dominance and control, and interpersonal complementarity theory provides an excellent explanation (Orford, 1986). Narcissistic traits are considered positively associated with dominance (Bradlee and Emmons, 1992). Therefore, dominant employees often do not work harmoniously with narcissistic leaders, thereby irritating relationship conflicts (Grijalva and Harms, 2014). Scholars have also emphasized future research to explore the relationship between narcissistic leaders and the personality traits of their employees (Nevicka et al., 2018a). In view of this situation, this research will investigate the moderating impact of dominant personality traits of employees on the previously established association between leader narcissism and supervisor-subordinate relationship conflict.

**FIGURE 1:**
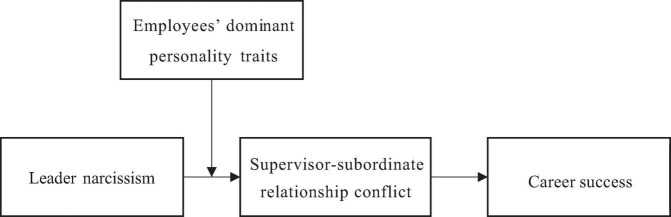
Theoretical model.

Our research contributes to the field of leadership and career success in multiple ways. First, this study expands the understanding of scholars regarding the influences of leader narcissism on the career success of employees, thereby enriching the field knowledge about distal outcomes. Second, our research clarifies how leader narcissism affects the career success of employees by exploring the mediating role of supervisor-employee relationship conflict. Third, this research advances our conceptualization of the leadership literature by indicating that the negative relationship between narcissistic traits in leadership roles and career success of employees can be moderated by the personality of the employee. The proposed theoretical model is presented in Figure 1.

## Theory and Hypotheses

### Leader Narcissism

As one of the Dark Triad traits, narcissism is characterized by a sense of grandiosity, a desire for power, a lack of sympathy, and an inflated self-view (Grijalva et al., 2015). Earlier studies have revealed that narcissism has a positive effect on leader emergence. It means that individuals with many narcissistic traits are more likely to become leaders due to their outgoing and glamorous personalities (Brunell et al., 2008; Grijalva et al., 2015). Narcissistic leadership occurs when the actions of leaders are motivated mainly by self-interest rather than being driven by the interests of an organization (Rosenthal and Pittinsky, 2006). Leader narcissism has been further divided into five parts, namely, charisma, self-interested influence, deceptive motivation, intellectual inhibition, and simulated consideration (Ouimet, 2010). Due to the higher power distance in Chinese cultures than Western cultures, it is also worth considering that there may be more narcissistic leaders in Chinese organizations (Liao et al., 2016).

The understanding of the influence of leader narcissism on workplace outcomes of employees has significantly broadened over the past decade. This trait comes as a mixed blessing. On the one hand, leader narcissism has been routinely associated with negative employee reactions such as counterproductive work behavior (Forsyth et al., 2012; Braun et al., 2018), employee time theft (Ding et al., 2018), and bad-mouthing (Carnevale et al., 2018). This leadership trait negatively impacts the voice of employees (Huang et al., 2020), pro-social behavior (Liu et al., 2017), and proactive behavior (Liao et al., 2019). Additionally, the affection and perception of employees are also influenced by narcissistic traits of their leaders, such that workers often report experiencing negative emotions and depression (Tokarev et al., 2017; Braun et al., 2018) as well as perceived victimization (ud din Khan et al., 2020) and perceived abusive supervision (Nevicka et al., 2018a). On the other hand, leader narcissism has also been shown positive effects on employees. For instance, when workers have fewer opportunities to observe their supervisor, perceived leadership effectiveness and job attitude of subordinates are positively correlated with leader narcissism (Nevicka et al., 2018b). Moreover, when leaders report high humility traits, narcissism positively impacts the job performance and engagement of subordinates (Owens et al., 2015). However, further investigation is still required to inform the literature on how leader narcissism relates to the career outcomes of employees and our understanding of antecedents to career success.

### Leader Narcissism and Career Success of Employees

Based on the interpersonal relationship perspective, a good supervisor-subordinate relationship means the chance to work-related autonomy and resources, which are the key to career success at the workplace (Seppälä et al., 2011; Guan and Frenkel, 2019). Previous research has illustrated that narcissistic individuals often have difficulty in maintaining interpersonal relationships due to a lack of trust and care for others (Ames et al., 2006). Extending this discussion, we contended that leader narcissism negatively affects the career success of employees. Specifically, narcissistic leaders often accuse, criticize, or attack their subordinates to show their superiority, thereon compromising the confidence of employees (Blair et al., 2017). Leaders of this type perpetuate their entitlement by exploiting subordinates and taking credit for the achievements of employees (Grijalva et al., 2015; Liu et al., 2017). These actions make subordinates feel as if they have less work autonomy, resulting in a loss of meaning in their work and a decrease in their internal work satisfaction. Collectively, these factors hamper the subjective career success of subordinate employees. Narcissistic leaders have even been shown to suppress the career development resources of employees due to a lack of interest in others and empathy (Rosenthal and Pittinsky, 2006), which can have significant consequences on the competitiveness of the worker within and outside the organization. The denial and blockage of work resource access by narcissistic leaders significantly hinder the objective career success of subordinate employees (Braun et al., 2018; Ding et al., 2018). In line with the reasoning outlined above, we predicted the following:

Hypothesis 1: Leader narcissism is negatively related to the career success of employees.

Hypothesis 1a: Leader narcissism is negatively related to the subjective career success of employees.

Hypothesis 1b: Leader narcissism is negatively related to the objective career success of employees.

### The Mediating Role of Supervisor-Subordinate Relationship Conflict

Empirical research supports acknowledging that relationship conflict in the workplace has detrimental effects on employees (Tepper et al., 2011). However, relationship conflict is considered an inescapable element of an organization (Kurtzberg and Mueller, 2005; Tillman et al., 2017). It is the manifestation of interpersonal incompatibility, causing individuals in conflicts to experience negative emotions (e.g., anger, distrust, fear, and frustration; Wall and Callister, 1995). In particular, supervisor-subordinate relationship conflict is regarded as a significant relationship conflict type, with a range of adverse employee outcomes, including decreased job performance (Jehn et al., 2010), organizational citizenship behaviors (Kacmar et al., 2012), and increased turnover intention (Tillman et al., 2017). Stemming from an interpersonal relationship perspective, leader narcissism negatively affects the supervisor-subordinate relationship, resulting in detrimental, cascading influences on the career success of employees (Xin and Pelled, 2003; Grijalva and Harms, 2014).

It is anticipated that leader narcissism is a likely cause of supervisor-subordinate relationship conflict. First, with an inflated self-view, self-interest, and reluctance to accept criticisms and differing opinions, narcissistic leaders often have difficulty in maintaining healthy relationships with others (Nevicka et al., 2011; Grijalva and Harms, 2014; Grijalva et al., 2015). These traits will impair the work interests of employees, thereby bringing the conflict in the relationship. Second, narcissistic leaders commonly exhibit negative behaviors (such as hostility, exploitation, and workplace violence) that can easily trigger supervisor-subordinate relationship conflict. Specifically, leader narcissism is so positively associated with hostility that individuals with more narcissistic traits are more likely to engage in physical and/or verbal conflict behaviors (Grijalva et al., 2015). Leaders with high narcissistic traits are also extremely exploitative, taking credit for the achievements of employees and being intolerant of shortcomings and failures of workers (Brummelman et al., 2016), behaviors that affect the interpersonal relationships. Critically, abusive and destructive behaviors of narcissistic leaders will break the bonds of trust within the relationship (Braun et al., 2018), thereby resulting in conflict. Third, leader narcissism has also a detrimental effect on the leader-member exchange, in which narcissistic leaders are more likely to be in relationship conflict with their subordinate employees (Liao et al., 2019).

Supervisor-subordinate relationship conflict is also expected to have a negative impact on the career success of employees. Subordinates in relationship conflict with their supervisors are more likely to experience negative emotions (Jehn, 1995), are less likely to have faith in their abilities, and receive less encouragement from their bosses (Xin and Pelled, 2003). Thereby, these factors reduce the job satisfaction (subjective career success) of employees. It is also important to consider that employees need to take extra efforts to deal with the situation above and beyond their normal duties when relationship conflict happens at the workplace. Thus, this can distract subordinate employees from essential work resources, lead to insufficient career development, and access to promotional resources (Tillman et al., 2017), which is in line with the conservation of resources theory (Hobfoll, 1989). At the same time, the moral exclusion theory (Opotow, 1990) suggests that leaders are more likely to morally exclude subordinates with whom they are experiencing relationship conflict (based on perceived fairness). Then, these behaviors negatively influence the objective career success of their employees and deprive the subordinate of essential career resources and professional development opportunities. Taking the state of the literature into account, we predicted the following:

Hypothesis 2: Supervisor-subordinate relationship conflict mediates the relationship between leader narcissism and career success of employees.

Hypothesis 2a: Supervisor-subordinate relationship conflict mediates the relationship between leader narcissism and subjective career success of employees.

Hypothesis 2b: Supervisor-subordinate relationship conflict mediates the relationship between leader narcissism and objective career success of employees.

### The Moderating Role of Dominant Personality Traits of Employees

Dominant personality traits describe tendencies toward agentic fundamental motives to influence, control, or gain mastery over the self, other people, and the environment (Horowitz et al., 2006). Individuals with a dominant personality enjoy the process of commanding or controlling others and get angry when their will is violated (Shechtman and Horowitz, 2006). Dominant employees are confident, effective, competitive, outspoken, and have a strong desire for power (Hong and Kaur, 2008). Interpersonal complementarity theory provides a useful framework for studying that how supervisor and subordinate characteristics mutually influence one another (Orford, 1986; Shechtman and Horowitz, 2006). For instance, when dominance is met by the other person submitting, it is more likely to lead to more satisfying and harmonious relationships. Alternatively, when dominant personalities are not balanced by submission, conflict can arise or be exacerbated.

It is anticipated that dominant personality traits of subordinate employees will moderate the relationship between leader narcissism and supervisor-subordinate relationship conflict. Specifically, previous studies have demonstrated positive correlations between narcissism and dominance traits (Bradlee and Emmons, 1992). Therefore, the interpersonal complementarity theory suggests that it is easy for disputes to arise between narcissistic leaders and dominant subordinates, with these personality incompatibilities further intensifying the relationship conflict. In a similar vein, dominant leaders tend to be less receptive to dominant employees because they feel threatened, which amplifies relationship conflict in these situations (Grant et al., 2011). On the contrary, employees with low-dominance traits are more submissive, less competitive, and willing to follow the actions of leaders (Hong and Kaur, 2008). Submissive subordinates tend to get along well with dominant leaders due to the detailed job duties and directions (Thoroughgood et al., 2012) and also tend to establish good rapports with narcissistic leaders, thereby further enhancing leader-subordinate relationship satisfaction (Grijalva and Harms, 2014). However, followers with high-narcissistic traits are more likely to be treated as outsiders by a narcissistic leader, which will often lead to more cases of relationship conflict (London, 2019). In summary, we postulated the following:

Hypothesis 3: Dominant personality traits of subordinate employees moderate the negative correlation between leader narcissism and supervisor-subordinate relationship conflict, such that the association is stronger for employees with more dominant personality traits and weaker for workers lower in personality dominance.

## Materials and Methods

### Participants and Procedures

Data were collected from employees in firms from China. Participation was voluntary, and all respondents were briefed on the academic purpose, procedure, anonymity, and confidentiality protocols of this study. Multiple waves (three-time points, with a lag of 2 months in each wave) of data collection were implemented. It has revealed that a 2-month lag is sufficient to reduce common method variances (Peng, 2013; Zhiqiang et al., 2020). At baseline (Time 1), 392 employees participated, providing demographic details (e.g., gender, education, and tenure) and ratings of narcissistic tendencies of their supervisor. Later, 364 of these employees returned valid questionnaires (92.86% response rate). Two months later (Time 2), questionnaires assessing dominance personality traits and supervisor-subordinate relationship conflict were distributed electronically to employees who completed the first round, and 327 surveys were returned with complete data (89.84% response rate). Time 3 questionnaires were distributed 2 months after Time 2, in which subjective and objective career success of employees was evaluated; 291 valid questionnaires were returned (88.99% response rate).

In the end, data from the final sample of 291 employees were included in the current analyses.

Demographic details are divulged in Table 1.

**TABLE 1:** Demographic details.

Items	Sections	Proportion
Gender	Male	54.98%
	Female	45.02%
Education	High school and below	1.72%
	College	9.62%
	Undergraduate	82.13%
	Master’s degree and above	6.53%
Tenure	Under 1 year	0.00%
	1–2 years	12.03%
	3–5 years	49.83%
	6–10 years	29.21%
	Over 10 years	8.93%
Position level	Entry-level position	30.24%
	Middle-level position	59.79%
	Deputy senior-level position	9.62%
	Senior-level position	0.35%
Enterprise nature	State-owned enterprise	38.83%
	Private enterprise	48.45%
	Foreign-funded enterprise	10.65%
	Others	2.07%

### Measures

Well-developed measurement scales were translated into Chinese using the translation and back-translation procedure (Brislin, 1986). All questionnaire items were measured using 5-point Likert scales ranging from 1 (strongly disagree) to 5 (strongly agree).

### Leader Narcissism

Self-reports often do not truly capture narcissistic traits due to personal sensitivities to the term *narcissism* (i.e., response bias). However, subordinate informants have been shown to provide reliable measures of the narcissistic traits of their leader. Therefore, the 10-item scale developed by Emmons (1987) was adopted, in which employees were asked to rate items such as “Your supervisor likes to be the center of attention” and “Your supervisor usually dominates any conversation.” The Cronbach’s α of this scale was 0.90.

### Career Success

The 5-item scale by Greenhaus et al. (1990) measured career satisfaction (subjective career success), including sample items such as “I am satisfied with the progress I have made toward meeting my overall career goals.” The 6-item scale by Eby et al. (2003) measured objective career success with items such as “I have many development opportunities in my organization.” The Cronbach’s α coefficient for career satisfaction was 0.89, and the coefficient for objective career success was 0.69.

### Supervisor-Subordinate Relationship Conflict

A 4-item scale developed by Landry and Vandenberghe (2009) evaluated supervisor-subordinate relationship conflict with questions such as “How much personality conflict is there between you and your supervisor?” Supervisor-subordinate relationship conflict yielded a Cronbach’s α coefficient of 0.81 in this sample.

### Dominant Personality Traits of Employees

The CAT-Personality Disorder Scales Static Form assessed dominant personality traits of employees with 6-items (e.g., “I have a strong need for power” and “I make demands on others”). This scale produced a Cronbach’s α coefficient of 0.86.

### Control Variables

Demographic variables such as gender, education, tenure, and position level were entered as control variables in all analyses because these factors impact career success (Ng et al., 2005). Likewise, enterprise nature was treated as a control variable, as it has been recognized as an essential factor to take into account in career success research (Spurk et al., 2019).

### Data Analyses

**FIGURE 2:**
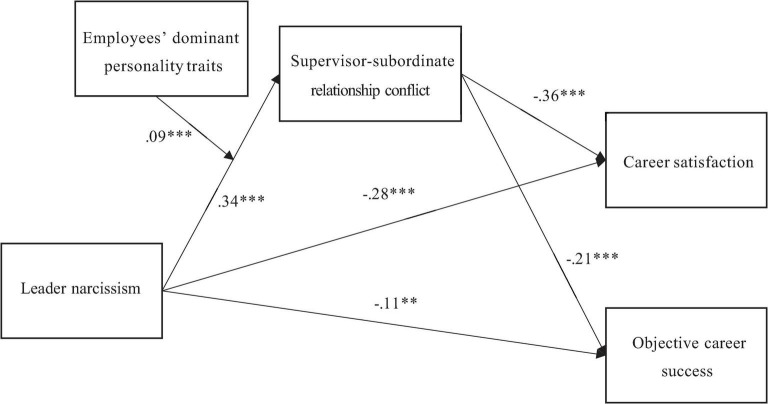
Path coefficients of the measurement model. Covariates were included in the model but are not presented for simplicity. ^**^p < 0.01 and ^***^p < 0.001.

Data analyses were implemented using SPSS 25 (Armonk, NY: IBM Corp) (IBM Statistics) and Mplus7.4 (Los Angeles, CA: Muthén & Muthén). As a preparatory analysis, Harman’s single-factor method (Harman, 1976) was used to check the common method variance of the data, and confirmatory factor analysis was applied to assess the discriminant validity of variables. In the main data analyses, first, descriptive statistics and correlation analyses were performed. Second, regression equations were applied to test the main effect of leader narcissism. Similarly, regression equations were executed to examine the mediation by supervisor-subordinate relationship conflict preliminarily. Additionally, the mediating effect was also assessed using 5,000 bootstrap estimates based on 95% bias-corrected CIs (PROCESS, model 4; Hayes, 2017). Finally, the moderating effect of dominant personality traits of employees was analyzed using the PROCESS macro (model 1; Hayes, 2017), and the interaction plot was constructed to show the details (Dawson, 2014). The path coefficients of the measurement model were calculated to clarify the data analyses (Figure 2).

## Results

### Common Method Variance Analysis

To avoid common method variance, we ensured employees could understand every item. For this purpose, we ran the questionnaire among a small sample of 25 employees to ensure that they perfectly understand the meaning of each item.

Harman’s single-factor method (Harman, 1976) was used to test common method variance. Specifically, a factor including all variables of this study was constructed, and exploratory factor analysis was performed. The non-rotating factor explained 30.4% of the variation (less than 40% of the total variation), indicating that it would not affect the research conclusion.

### Confirmatory Factor Analysis

Confirmatory factor analysis was applied to test the discriminant validity of leader narcissism, supervisor-subordinate relationship conflict, employee dominance, subjective career success (career satisfaction), and objective career success (Table 2). The five-factor model was preferable, signifying that the study variables provide good discriminant validity.

**TABLE 2:** Confirmatory factor analysis results.

Models	χ^2^	df	χ^2^/df	CFI	TLI	RMSEA
Five-factor model (LN; SSRC; CS; OCS; DPT)	627	314	2.00	0.91	0.90	0.06
Four-factor model (LN; SSRC; DPT; CS+OCS)	685	318	2.15	0.90	0.89	0.06
Three-factor model (LN; SSRC+DPT; CS + OCS)	1116	321	3.48	0.79	0.76	0.09
Two-factor model (LN; SSRC+CS+OCS+DPT)	1523	323	4.72	0.67	0.64	0.11
One-factor model (LN+SSRC+CS+OCS+DPT)	1881	324	5.81	0.57	0.54	0.13

*LN, leader narcissism; SSRC, supervisor-subordinate relationship conflict; CS, career satisfaction (subjective career success); OCS, objective career success; DPT, dominant personality traits; CFI, comparative fit index; TLI, Tucker-Lewis index; RMSEA, root mean square error of approximation.*

### Correlation Analyses

Descriptive and inferential statistics are shown in Table 3. Leader narcissism was positively associated with supervisor-subordinate relationship conflict (*r* = 0.54 and *p* < 0.01), but negatively related to career satisfaction (*r* = −0.54 and *p* < 0.01) and objective career success (*r* = −0.34 and *p* < 0.01). These results provide preliminary support for Hypotheses 1a and 1b.

**TABLE 3:** Means, SDs, and correlations of variables.

Variables	*M*	*SD*	1	2	3	4	5	6	7	8	9
1. Gender	1.55	0.50									
2. Education	2.94	0.48	0.09								
3. Tenure	3.35	0.81	−0.18**	–0.08							
4. Position level	1.80	0.61	−0.15*	0.17**	0.31**						
5. Enterprise nature	1.76	0.72	0.06	−0.16**	–0.09	−0.13*					
6. LN	2.14	0.81	–0.06	–0.02	–0.02	–0.04	0.03				
7. SSRC	1.61	0.52	–0.12	0.08	–0.11	–0.08	–0.02	0.54**			
8. DPT	2.57	0.74	0.06	–0.04	–0.04	–0.01	0.07	0.28**	0.23**		
9. CS	4.08	0.60	0.01	0.02	0.01	0.08	–0.03	−0.54**	−0.50**	−0.14*	
10. OCS	3.93	0.43	0.04	0.08	–0.05	0.15*	0.02	−0.34**	−0.35**	0.03	0.59**

LN, leader narcissism; SSRC, supervisor-subordinate relationship conflict; DPT, dominant personality traits; CS, career satisfaction; OCS, objective career success; n = 291. *p < 0.05; **p < 0.01.

### Hypothesis Testing

#### Main Effect of Leader Narcissism

Hypotheses 1a and 1b posit that leader narcissism is negatively related to subjective career success (career satisfaction) and objective career success of employees. These hypotheses were tested further using regression equations (Table 4). Model 2 supported Hypothesis 1a, that is, leader narcissism is negatively and significantly associated with the career satisfaction of employees (β = −0.40 and *p* < 0.001). Likewise, Model 4 shows that leader narcissism negatively predicted the objective career success of employees (β = −0.18 and *p* < 0.001), supporting Hypothesis 1b.

**TABLE 4:** Regression analysis models.

Variables	SSRC	CS		OCS	
	Model 1	Model 2	Model 3	Model 4	Model 5
Gender	−0.12*	–0.01	–0.06	0.02	–0.01
Education	0.11	–0.01	0.03	0.04	0.06
Tenure	–0.06	–0.02	–0.04	–0.05	−0.06*
Position level	–0.05	0.07	0.05	0.11**	0.10*
Enterprise nature	–0.02	–0.01	–0.01	0.03	0.02
LN	0.34***	−0.40***	−0.28***	−0.18***	−0.11**
SSRC			−0.36***		−0.21***
*R* ^2^	0.32	0.30	0.37	0.15	0.19

LN, leader narcissism; SSRC, supervisor-subordinate relationship conflict; CS, career satisfaction; OCS, objective career success. n = 291. *p < 0.05; **p < 0.01; ***p < 0.001.

#### Mediation by Supervisor-Subordinate Relationship Conflict

Hypothesis 2 posits that supervisor-subordinate relationship conflict would mediate the relationship between leader narcissism and career success of employees. Therefore, the direct effect of leader narcissism on supervisor-subordinate relationship conflict (*a* path) and the indirect effect of supervisor-subordinate relationship conflict on the career success of employees in the presence of leader narcissism (*b* path) was evaluated (Table 4). Model 1 reveals that leader narcissism is positively related to supervisor-subordinate relationship conflict (β = 0.34 and *p* < 0.001). Furthermore, Model 3 divulges that supervisor-subordinate relationship conflict is negatively associated with subjective career success of employees (i.e., career satisfaction; β = −0.36 and *p* < 0.001) in the presence of leader narcissism, which become lesser in magnitude (β = −0.28 and *p* < 0.001). Similarly, Model 5 shows that supervisor-subordinate relationship conflict is negatively related to the objective career success of employees (β = −0.21 and *p* < 0.001) in the presence of leader narcissism, attenuating in both magnitude and significance (β = −0.11 and *p* < 0.01). These patterns are consistent with Hypotheses 2a and 2b.

To formally test the mediation effect of supervisor-subordinate relationship conflict, a bootstrapping procedure based on the PROCESS macro (Hayes, 2017) was used to estimate the indirect, direct, and total effects as well as 95% CIs. Table 5 shows that the degree of significance observed regarding the influence of leader narcissism on the two measures of career success of employees can be attributed to its indirect effects through supervisor-subordinate relationship conflict rather than its direct effects. Specifically, the indirect effects of leader narcissism on career satisfaction of employees [estimate = −0.12; 95% CI = (−0.22, −0.04)] and objective career success [estimate = −0.07; 95% CI = (−0.13, −0.03)] through supervisor-subordinate relationship conflict are significant. Therefore, Hypotheses 2a and 2b are supported.

**TABLE 5:** Mediation effect analysis.

Models	Indirect effect [(95% BootLLCI, BootULCI)]	Direct effect [(95% BootLLCI, BootULCI)]	Total effect [(95% BootLLCI, BootULCI)]
LN-SSRC-CS	−0.12 [(−0.22, −0.04)]	−0.28 [(−0.44, −0.13)]	−0.40 [(−0.54, −0.27)]
LN-SSRC-OCS	−0.07 [(−0.13, −0.03)]	−0.11 [(−0.18, −0.03)]	−0.18 [(−0.25, −0.11)]

*LN, leader narcissism; SSRC, supervisor-subordinate relationship conflict; CS, career satisfaction; OCS, objective career success. Sample n = 291; bootstrap n = 5,000.*

#### Moderation by Dominant Personality Traits of Employees

**FIGURE 3:**
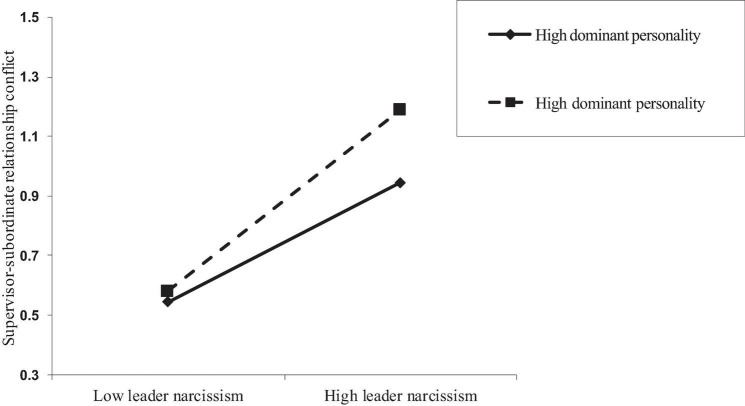
The moderating effect of dominant personality traits of employees on the relationship between leader narcissism and supervisor-subordinate relationship conflict.

Leader narcissism and dominant personality traits of employees were mean-centered before entering the PROCESS macro to examine the moderating effect of the dominant personality of employees (Hypothesis 4). Table 6 presents the results for the conditional effect of leader narcissism on supervisor-subordinate relationship conflict at low and high values (±1 SD from the mean) of dominant personality traits of employees. The effect of leader narcissism on supervisor-subordinate relationship conflict was weak at low levels of dominant personality traits of employees [−1 SD from the mean: estimate = 0.21; 95% CI = (0.12, 0.29)] but was substantially stronger at high levels of dominant personality traits of employees [+1 SD from the mean: estimate = 0.42; 95% CI = (0.34, 0.51)], supporting Hypothesis 4. An interaction plot was constructed following the procedures recommended by Dawson (2014; Figure 3).

**TABLE 6:** Moderation effect analysis.

	Effect	SE	LLCI	ULCI
Low DPT (−1 SD)	0.21	0.05	0.12	0.29
Mean DPT	0.31	0.03	0.25	0.38
High DPT (+1 SD)	0.42	0.04	0.34	0.51

*DPT, dominant personality traits.*

## Discussion

Based on the interpersonal relationship perspective, the influence of leader narcissism on employee career success was empirically investigated, which were supervisor-subordinate relationship conflict mediation and employee dominance trait moderation. Results indicate that leader narcissism negatively influences subjective and objective career success of employees, consistent with the conclusion of previous studies. Many scholars have demonstrated that leader narcissism has detrimental effects on employee workplace outcomes. For example, leader narcissism decreases proactive behavior (Liao et al., 2019) and voice (Huang et al., 2020) of employees. In this study, we added to this body of literature by demonstrating that supervisor-subordinate relationship conflict partially mediates the relationship between leader narcissism and career success of employees, opposing the conclusion of Volmer et al. (2016) that leader narcissism advances career success of employees. The contradiction may be that narcissistic leaders tend to retain loyal and submissive employees to gain sustained praise from them. This study enriches this conclusion, indicating that the positive relationship between leader narcissism and career success of employees will only occur when the supervisor and subordinate employee get along well. However, when they conflict, the opposite effect may occur. This research also indicates that dominant personality traits of employees moderate the association between leader narcissism and supervisor-subordinate relationship conflict. According to the previous results reported throughout the literature, submissive subordinates can maintain good relationships with narcissistic leaders, while dominant subordinates are more likely to have conflicts with leaders of this type (Grijalva and Harms, 2014; London, 2019).

### Theoretical Implications

The conclusion of this study has several theoretical implications. First, this research empirically demonstrates that the detrimental influence leader narcissism has on the career success of employees, enriching the literature on the workplace outcomes of leader narcissism. Although scholars have made great progress in researching the consequences of leader narcissism (e.g., counterproductive work behavior, Braun et al., 2018; employee proactive behavior, Liao et al., 2019), these studies have focused primarily on the proximal outcomes of leader narcissism, paying less attention to distal outcomes such as career success of employees. Thus, this research deepens the understanding of the potential long-term impacts of leader narcissism of the field.

Second, this research explores the influence of leadership on the career success of employees from a new perspective. Previous studies mainly focused on career success from the perspective of the self-concept of employees (Joo and Lim, 2013; Chughtai, 2018). For the first time, we have uncovered the impact of leader narcissism on career success from the interpersonal relationship perspective, laying a theoretical foundation for preventing the adverse effects of narcissistic leadership.

Moreover, this study extends the understanding of the effects of leader narcissism in light of differences in personality traits of subordinate employees. On the basis of the interpersonal complementarity theory, this study analyzes the moderating effect of dominant personality traits, thereby advancing our conceptualization of the leadership literature.

### Practical Implications

The findings of this study also have several practical implications. First, this research can be helpful for organizations looking to understand more about the harm of leader narcissism and to take effective measures to constrain its negative effects. Moreover, it highlights the adverse effects of leader narcissism on the career success of their employees. To reconcile this problem, organizations can restrict the power of narcissistic leaders through institutional design and recruitment processes. Second, this research can be helpful for managers seeking to promote the career success of employees more effectively by striving to establish and maintain good relationships between supervisors and subordinate workers. Third, these findings can be beneficial for narcissistic leaders, since they can learn to improve adaptability to different personalities in the workplace and seek employees that best suit their supervisory and personality styles. In particular, the moderating role of dominant personality traits suggests that organizations can implement personality assessments and classification as part of the hiring and interview process as well as ongoing management of employees. These methods would allow organizations and managers to match better supervisors with subordinate employees based on personality compatibility.

### Limitations and Future Directions

Several limitations are worth noting in this study. First, all the questionnaires were rated by employees. Although the common method bias does not present a major issue in and of itself, the congruency of responses from the perspectives of supervisors is lacking. Therefore, we hoped that there will be some improvement in future research by using multiple informants. Second, the measurement tools used to assess narcissism are currently not unified in the literature (Ding et al., 2018; Nevicka et al., 2018a), resulting in some deviation in results across different measurement scales. Thus, the present results need to be further verified with other scales, such as the NPI-16 (Ames et al., 2006) or scales of narcissism by Hochwarter and Thompson (2012). Finally, this study only examines the relationship between leader narcissism and career success of employees from the interpersonal relationship perspective, focusing on the mediating role of supervisor-subordinate relationship conflict. However, it can also be conducted from other perspectives, such as the resource perspective.

## Data Availability Statement

The original contributions presented in the study are included in the article/supplementary material, further inquiries can be directed to the corresponding author/s.

## Ethics Statement

Written informed consent was obtained from the individual(s) for the publication of any potentially identifiable images or data included in this article.

## Author Contributions

HW designed the research and revised the whole manuscript. DL analyzed the data and wrote and revised the manuscript. LW and ZD collected the data and discussed the results. All authors contributed to the article and approved the submitted version.

## Conflict of Interest

The authors declare that the research was conducted in the absence of any commercial or financial relationships that could be construed as a potential conflict of interest.

## Publisher’s Note

All claims expressed in this article are solely those of the authors and do not necessarily represent those of their affiliated organizations, or those of the publisher, the editors and the reviewers. Any product that may be evaluated in this article, or claim that may be made by its manufacturer, is not guaranteed or endorsed by the publisher.
